# Distribution pattern and molecular signature of cholinergic tuft cells in human gastro-intestinal and pancreatic-biliary tract

**DOI:** 10.1038/s41598-019-53997-3

**Published:** 2019-11-25

**Authors:** Burkhard Schütz, Anna-Lena Ruppert, Oliver Strobel, Michael Lazarus, Yoshihiro Urade, Markus W. Büchler, Eberhard Weihe

**Affiliations:** 1 Institute of Anatomy and Cell Biology, Philipps-University, Marburg, Germany; 2 Department of General Surgery, University of Heidelberg, Heidelberg, Germany; 3 European Pancreas Center, University of Heidelberg, Heidelberg, Germany; 4 International Institute for Integrative Sleep Medicine (WPI-IIIS), University of Tsukuba, 1-1-1 Tennodai, Tsukuba, Ibaraki 305-8575 Japan; 5 Isotope Science Center, The University of Tokyo, 2-11-16 Yayoi, Bunkyo-ku, Tokyo 113-0032 Japan; 6 Graduate School of Pharmaceutical Sciences, Kitasato University, 5-9-1 Shirokane, Minato-ku, Tokyo 108-8641 Japan

**Keywords:** Gastroenterology, Cell signalling

## Abstract

Despite considerable recent insight into the molecular phenotypes and type 2 innate immune functions of tuft cells in rodents, there is sparse knowledge about the region-specific presence and molecular phenotypes of tuft cells in the human digestive tract. Here, we traced cholinergic tuft cells throughout the human alimentary tract with immunohistochemistry and deciphered their region-specific distribution and biomolecule coexistence patterns. While absent from the human stomach, cholinergic tuft cells localized to villi and crypts in the small and large intestines. In the biliary tract, they were present in the epithelium of extra-hepatic peribiliary glands, but not observed in the epithelia of the gall bladder and the common duct of the biliary tract. In the pancreas, solitary cholinergic tuft cells were frequently observed in the epithelia of small and medium-size intra- and inter-lobular ducts, while they were absent from acinar cells and from the main pancreatic duct. Double immunofluorescence revealed co-expression of choline acetyltransferase with structural (cytokeratin 18, villin, advillin) tuft cell markers and eicosanoid signaling (cyclooxygenase 1, hematopoietic prostaglandin D synthase, 5-lipoxygenase activating protein) biomolecules. Our results indicate that region-specific cholinergic signaling of tuft cells plays a role in mucosal immunity in health and disease, especially in infection and cancer.

## Introduction

Tuft cells represent a minor sub-population of post-mitotic epithelial cells in the mucosal lining of the mammalian alimentary tract^1,2^. On the morphological level, tuft cells are characterized by an apical tuft of stiff microvilli^3^. On the molecular level they show striking similarities with taste cells in the oral cavity. Hence, gastro-intestinal tuft cells have been shown to express structural markers such as villin^4^, advillin^5^, cytokeratin-18 (CK18)^6^ and doublecortin-like kinase 1 (DCLK1)^7^; taste receptors, such as sweet/umami (T1Rs) and bitter taste receptors (T2Rs)^8,9^; components of the canonical taste transduction cascade, i.e. α-gustducin^10,11^, phospholipase C β2 (PLCß2)^12^, and transient receptor potential isoform M5 (TRPM5)^13^; and enzymes for prostaglandin and leukotriene production, namely cyclooxygenases 1 (COX1 or Ptgs1) and 2 (COX2 or Ptgs2), hematopoietic prostaglandin-D-synthase (HPGDS)^5,14^, and arachidonate 5-lipoxygenase (ALOX5)^5^. The development of tuft cells depends on the transcription factor POU class 2 homeobox 3 (POU2F3)^15^. Only very recently, diverse functions of gastro-intestinal tuft cells in the context of cancer, infection, and inflammation have begun to emerge^16^.

Gastro-intestinal tuft cells, like classical gustatory cells in oral taste buds, are presumed secondary chemosensory cells, implying that upon activation they signal either onto a sensory nerve ending, or onto resident and/or mobile cells in their local neighborhood, or both. While gustatory cells in the oral cavity express and release a plethora of neuroactive signaling substances^17,18^, gastro-intestinal tuft cells may utilize certain inflammation- and neuron-related molecules, including prostaglandins and leukotrienes (see above), the cytokine interleukin (IL) 25^19^, but also acetylcholine (ACh). ACh is produced from choline and acetyl-CoA by the enzyme choline acetyltransferase (ChAT). In mice, subsets of taste cells in the oral cavity contain ChAT-immunoreactivity^20^. Also extra-oral tuft cells^21^, and some functionally related chemosensory cells in the respiratory^22–24^ and urinary tracts^25,26^ express a cholinergic phenotype. Transgenic mice that produce enhanced green fluorescent protein (EGFP) under the control of the ChAT promoter^27,28^ are useful tools to visualize cholinergic tuft cells. It is still under debate, however, if classical taste and gastro-intestinal tuft cells accumulate and store ACh in secretory vesicles, and thus require the vesicular acetylcholine transporter (VAChT), as do cholinergic neurons^29^. Previously, we could show that almost all gastro-intestinal tuft cells in mice contain mRNA and immunoreactivity for ChAT, but not for VAChT, with the exception of some VAChT immunoreactive cells in the ascending colon^21^.

A presence of cells with tuft cell-like morphologies has been reported for the human gastro-intestinal tract^30–32^, and evidence for the presence of ACh and enzymatic activity of ChAT in gut surface epithelia has been provided^33,34^. Furthermore, ChAT immunoreactivity was detected in solitary cells in the epithelia of both the small and large human intestines. The staining pattern, however, suggested an enteroendocrine nature of these solitary ChAT cells in the epithelium^35,36^, or that they represent a yet undefined cell population^37^. Equivalent to mice, VAChT mRNA expression was absent throughout the epithelium of human gut mucosa^38^. This is indicative of an independent expression of the two components of the so-called cholinergic gene locus^29^ in non-neuronal cells of the human gut epithelium.

Despite of few reports identifying cells with a prominent apical tuft in extrahepatic human gall bladder and bile duct epithelia on the ultrastructural level^32,39^, the cholinergic and additional molecular signatures of tuft cells in the normal human biliary tract are still unexplored. Similarly, although the presence of a tuft cell population in the normal human pancreatic duct system has been reported^40^, a possible cholinergic co-phenotype is unknown.

Here, we present the first immunohistochemical characterization of the presence, distribution, and molecular phenotypes of cholinergic tuft cells in the normal human alimentary tract, including intestinal, biliary and pancreatic systems. Double immunofluorescence analysis of the cholinergic marker, ChAT, with structural, immune signaling, and enteroendocrine markers unambiguously identified the tuft cell as the sole cellular source for epithelial biosynthesis of ACh in the human alimentary tract and suggests that cholinergic tuft cell signaling may play a central role in mucosal immunity in health and disease, especially in infection and cancer.

## Results

### Segment-specific distribution and shape of cholinergic tuft cells in the human gastro-intestinal tract mucosal epithelia

**Figure 1:**
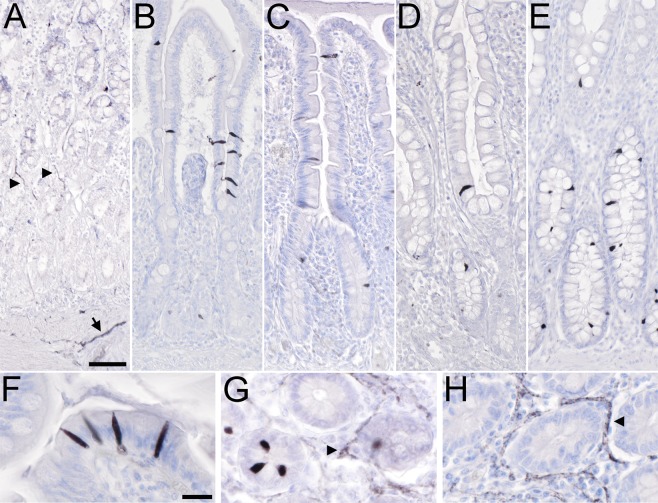
Distribution of non-neuronal ChAT-immunoreactive cells in the human gastro-intestinal tract mucosal epithelia. (**A**) In the stomach, ChAT-immunoreactive (ir) nerve fibers trespass the mucosal glands (arrowheads) and the muscle layers (arrow). Epithelial cell body staining is not detectable. (**B**) In the duodenal mucosa, intra-epithelial cells are ChAT-positive. They locate to both, villi and crypts. (**C**) A similar distribution pattern is found in the jejunum. (**D**) In the ileum, ChAT-ir cells mostly locate to the lower third of the villi and to the crypts, while they are mostly absent from the upper two thirds of the villi. (**E**) In the colon, ChAT-ir cells primarily locate to the lower half of the crypts while they are almost absent from the upper half. (**F**) ChAT-ir cell morphology is similar in all aspects of the intestine. Mainly in villi, they are slim cells with an oval shape, and span the entire epithelium. A protrusion on the apical side extends into the mucosal brush border. (**G + H**) While ChAT immunoreactivity labels intra-epithelial cells and nerve fibers (arrowhead in **G**), VAChT-ir is restricted to nerve fiber plexus (arrowhead in **H**), shown here for the jejunum. All sections were counter-stained with hemalaun. For ChAT detection, the goat-anti-ChAT antiserum was used. The bar in A equals 50 µm and applies to (**A–E**). The bar in (**F**) equals 20 µm and applies to (**F–H**).

We first analyzed by single brightfield immunohistochemistry the presence and distribution of non-neuronal cholinergic cells in the normal human gastro-intestinal tract, using the goat-anti-ChAT antiserum as marker. In all our stomach samples, we observed ChAT-immunoreactive (ir) nerve fibers innervating the mucosal glands (Fig. 1A). Also, neuronal cell bodies in the myenteric plexus were ChAT-ir (data not shown). In contrast, ChAT-ir epithelial cells were not present. In the duodenum (Fig. 1B) and in the jejunum (Fig. 1C), ChAT-immunoreactivity was seen in solitary epithelial cells scattered along the crypt-villus units (cvu). Single ChAT-ir cells were also present in the mucosal outflow tracts of duodenal Brunner´s glands, but were absent from deeper submucosal glandular epithelium (data not shown). In the ileum, ChAT-ir cells were predominantly found in the lower parts of the villi and in the crypt epithelium, but were largely absent from upper villus parts (Fig. 1D). Similarly, ChAT-ir epithelial cells were restricted to about the lower third of the colonic crypts (Fig. 1E). Such distribution patterns were true for all samples analyzed. ChAT-ir cells represented a rare subpopulation in the mucosal lining of the gut, with 1.6 ± 0.02 cells per cvu in duodenum, 1.5 ± 0.38 cells per cvu in the jejunum, 1.1 ± 0.54 cells per cvu in the ileum, and 1.1 ± 0.15 cells per cvu in the colon (*n* = 50–70 cvu analyzed per specimen).

ChAT-ir cell morphology was similar in all divisions of the intestine. In villus parts they appeared flat-oval with long and slim basal and apical protrusions, and spanned the entire epithelium (Fig. 1F). Mainly in crypts, protrusions were short and cells more flask-shaped (Fig. 1G). The cholinergic phenotype of these cells was restricted to the expression of ChAT, since immunoreactivity for the vesicular acetylcholine transporter, VAChT, was absent (Fig. 1H).

### Distribution of cholinergic tuft cells in human extrahepatic bile duct and in the pancreatic ductal system

**Figure 2:**
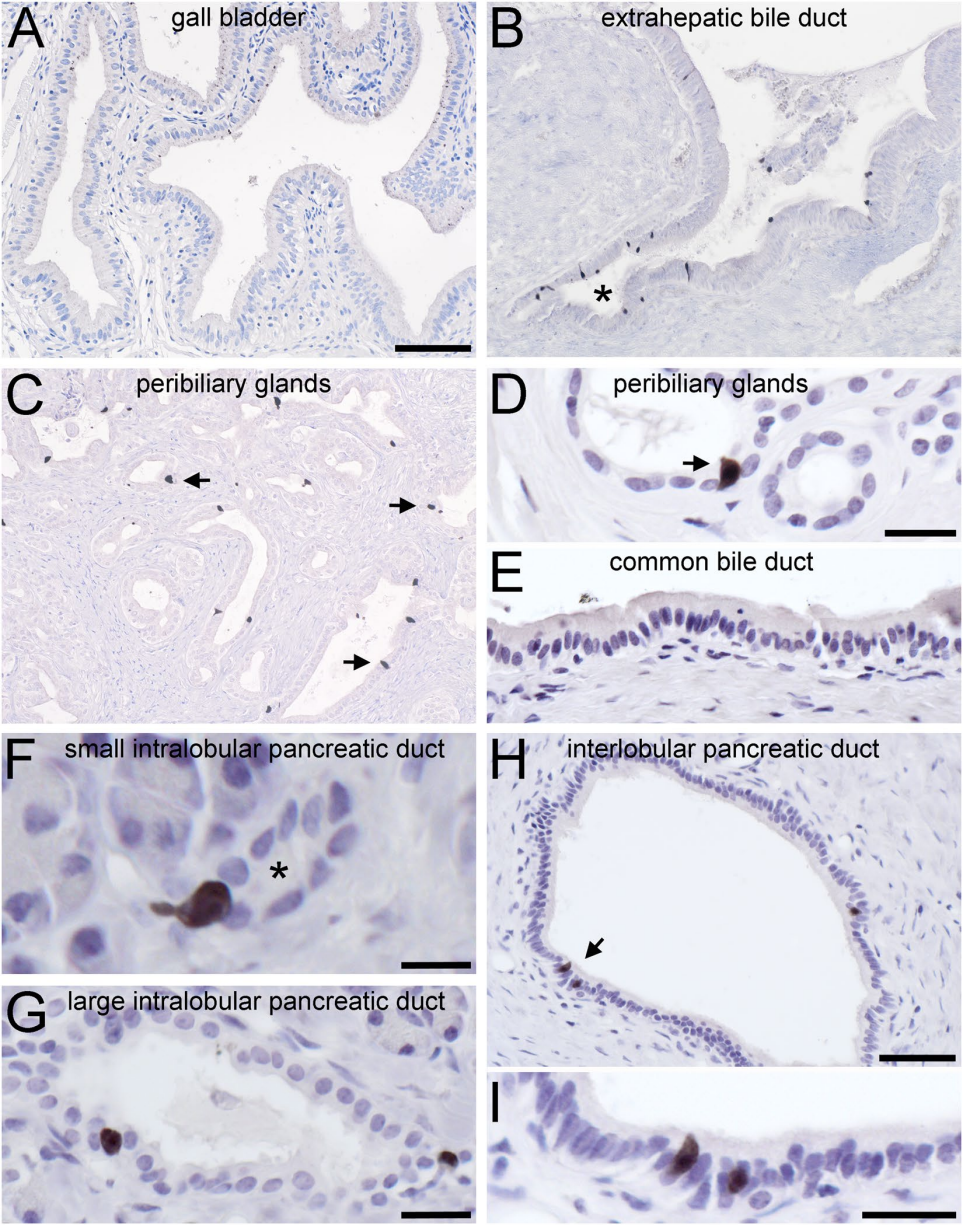
Presence and distribution of ChAT-immunoreactive cells in human extrahepatic biliary and pancreatic ducts. (**A**) ChAT immunoreactive (ir) cells are absent from the main gall bladder epithelium, but (**B**) are present at the exit sites of extrahepatic peribiliary glands (marked by asterisk). (**C**) Solitary ChAT-ir cells display a scattered distribution in the walls of extra-hepatic peribiliary glands. (**D**) The morphology of such a flask-shaped cell (marked by arrow) in higher magnification. (**E**) ChAT-ir cells are absent from the wall of the common bile duct. (**F**,**G**) ChAT-ir cells are numerous in small (**F**, labeled by asterisk) and medium (**G**) diameter intralobular pancreatic ducts. (**H**,**I**) In the wall of interlobular pancreatic ducts they are found as solitary cells (marked by arrow in **H**, and magnified in **I**), but are absent from the common pancreatic duct (not shown). For ChAT detection, the goat-anti-ChAT antiserum was used. The bar in **A** equals 100 µm and also applies to **B and C**. The bar in (**D)** equals 20 µm and also applies to **E** and **G**. The bar in (**F)** equals 10 µm, in (**H**) equals 50 µm, and in (**I**) equals 20 µm.

In mice, numerous ChAT-ir cells reside in the epithelial lining of the gall bladder, the cystic duct, the common bile duct, and the papilla of Vater^21^. In all our human samples, however, ChAT-ir cells were absent from the simple columnar gall bladder epithelium (Fig. 2A). Instead, solitary, slim or flask-shaped ChAT-ir cells were present in the extra-hepatic peribiliary gland columnar (Fig. 2B,C) and cuboidal (Fig. 2D) epithelia, but were absent from the common bile duct (Fig. 2E) Again, this distribution pattern was true for all specimens analyzed.

In the exocrine pancreas, solitary ChAT-ir cells lined cuboidal small- (Fig. 2F) and large-sized (Fig. 2G) intralobular, and columnar interlobular (Fig. 2H,I) ducts, but were absent from acinar cells, from the common pancreatic duct, and from the islets of Langerhans, the endocrine part of the pancreas (data not shown). Also, ChAT-ir cells in bile and pancreatic ducts did not display VAChT expression in all specimens analyzed (data not shown).

### Human cholinergic tuft cells exhibit region-specific combinatorial expression patterns of structural and biomolecule tuft cell markers

**Figure 3:**
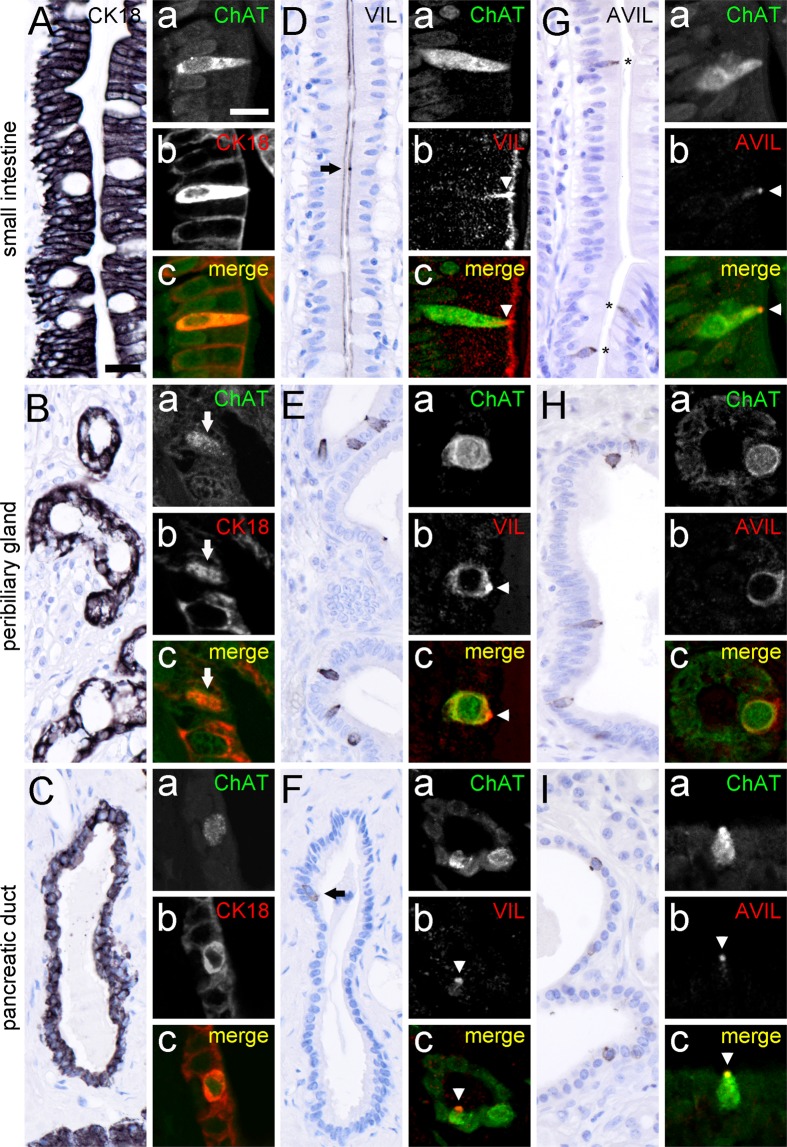
Presence of tuft cell morphology markers in ChAT-immunoreactive tuft cells. Cytokeratin 18 (CK18) immunoreactivity is present in many, if not all epithelial cells from the small intestine (**A**), peribiliary glands (**B**), and pancreatic ducts (**C**). Hence, double-immunofluorescence analysis of ChAT with CK18 revealed complete co-expression in human small intestine (**A**a–c), peribiliary glands (**B**a–c, arrow points to ChAT/CK18 co-immunoreactive cell), and pancreatic ducts **(C**a–c). Villin (VIL) immunoreactivity is present at the apical brush border of almost all intestinal epithelial cells (**D**). Local accumulations indicate location of tuft cells (arrow in **D**), which is confirmed by double labeling of ChAT with VIL (Da–c, arrowhead points to VIL accumulation in tuft cell brush border). VIL immunoreactivity is also present in solitary cells in extrahepatic peribiliary glands (**E**) and in pancreatic ducts (**F**). Again, ChAT immunoreactivity is present in VIL immunoreactive cells (**E**a–c, and **F**a–c); arrowheads demarcate VIL immunoreactivity in apical tuft). Advillin (AVIL) immunoreactivity is restricted to solitary cells in the epithelial lining of the small intestine (**G**, asterisks), peribiliary glands (**H**), and pancreatic ducts (**I**). In all these locations AVIL co-localized to ChAT (**G**a–c, **H**a–c, **I**a–c). The single antibody labeling (a,b) is shown in greyscale, the composite (c) in green (ChAT) and in red (CK18, VIL, AVIL) color coding, respectively. For all double-immunofluorescent analyses of ChAT, the goat-anti-ChAT antiserum was used. The bar in **A** equals 20 µm and applies to all bright-field images. The bar in **Aa** equals 10 µm and applies to all images from immunofluorescence analysis.

Having established the distinct presence of ChAT-ir epithelial cells in the human intestinal, in the extrahepatic biliary and in the exocrine pancreatic systems, we asked whether these cells, like in rodents, represent tuft cells. Murine tuft cells are discriminated from other cells in the respective epithelia by their strong expression of certain structural proteins, e.g. cytokeratin 18 (CK18) and villin. Both in rat^6^ and in mouse^21^ CK18 is expressed by a major subpopulation of mucosal epithelial cells, and labeling is more intense in tuft cells compared to non-tuft cells. In our samples taken from the human intestine (Fig. 3A), accessory bile ducts (Fig. 3B), and pancreatic ducts (Fig. 3C), CK18 antibodies strongly labeled the entire epithelia. Double immunofluorescent analysis revealed an overlap of ChAT-ir cells with the strongly labeled CK18 cells (Fig. 3A–Ca–c).

Villin, an actin filament crosslinking protein, is highly expressed in the apex of tuft cells, including the apical brush border^4,14^. In the human intestine, villin expression was found on the luminal side of many, if not all cell types along the one-layered epithelium, exemplified here for the small intestine (Fig. 3D). Solitary cells displaying a strong apical accumulation of villin-ir were also ChAT-ir (Fig. 3Da–c). In both, peribiliary glands and pancreatic ducts, villin-ir was found restricted to solitary cells (Fig. 3E,F). In addition to the apical brush border, also the lateral cell aspects were stained. All these villin-ir cells were co-positive for ChAT-ir.

Advillin is another member of the gelsolin/villin family, and structurally related to villin^41^. In mice, advillin is expressed by many sensory and autonomic neurons^42^, but also found in taste cells in lingual taste buds^43^. In the human intestine, advillin-ir is believed to be present in endocrine cells (www.proteinatlas.org). We observed that advillin expression was not pan-epithelial, when compared to villin, but restricted to the solitary ChAT-ir cells in the human intestine (Fig. 3G). Likewise, advillin staining in the human peribiliary glands (Fig. 3H), and pancreatic duct epithelia (Fig. 3I) was found confined to ChAT-expressing tuft cells.

The serine/threonine-protein kinase, doublecortin-like kinase 1 (Dclk1), is an established tuft cell marker in the mouse alimentary tract^7,44^, but recently was described to be restricted to a subset of enteroendocrine cells in the human intestine^45^. Using the same antiserum, we could confirm that DCLK1 is expressed by ChAT-ir tuft cells in the mouse gastro-intestinal system (data not shown). In our human gut samples, however, DCLK1-ir did not co-localize with ChAT-ir. Instead, DCLK1-ir co-localized with chromogranin A (CGA)-ir enteroendocrine cells. This staining, however, was interpreted to be non-specific since it could not be blocked when pre-incubating the primary DCLK1 antiserum with an excess of the peptide used for immunization (data not shown).

**Figure 4:**
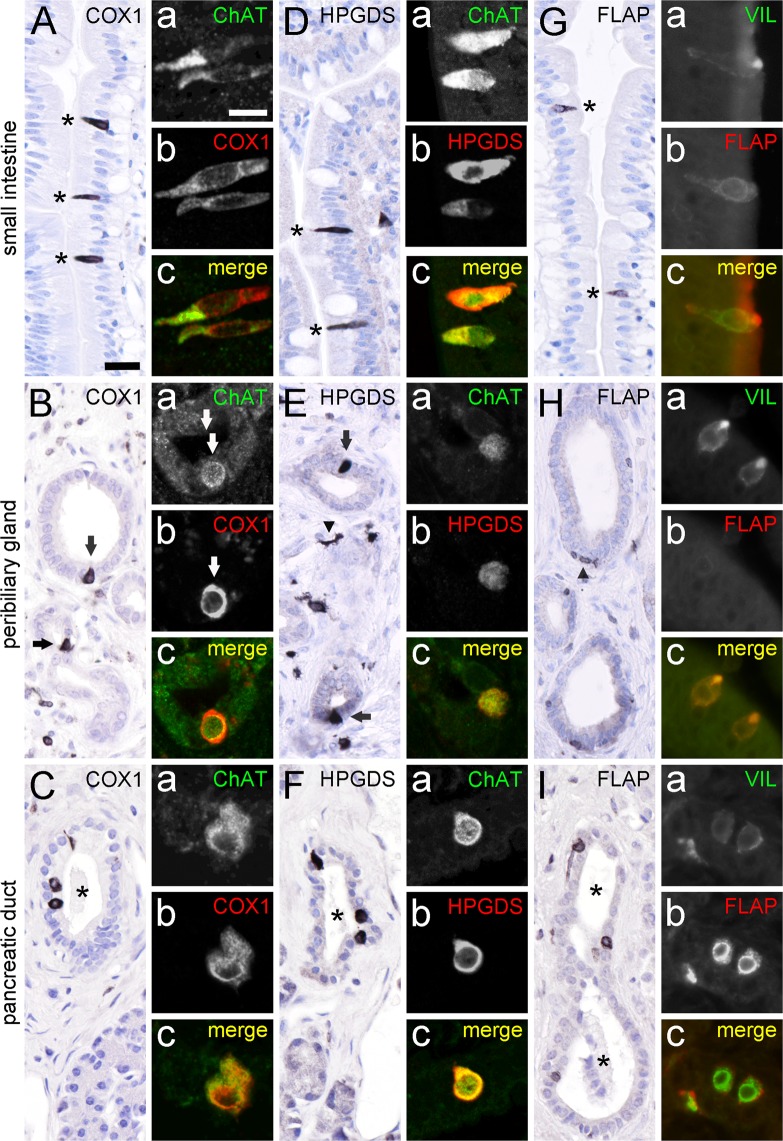
ChAT-immunoreactive tuft cells express proteins for prostaglandin and leukotriene production. COX1-ir is present in solitary cells in the human small intestine mucosal epithelium (**A**, marked with asterisks), in accessory bile ducts (**B**, marked with arrows), and in pancreatic ducts (**C**, duct marked by asterisk). Double-immunofluorescence analysis revealed complete co-expression of ChAT (rabbit-anti-ChAT antiserum) with COX1 in epithelial cells (**A**a–c, **B**a–c, and **C**a–c). HPGDS-ir is present in solitary cells in the human small intestine mucosal epithelium (**D**; marked with asterisks), in peribiliary glands (**E**, marked with arrows), and in pancreatic ducts (**F**, duct marked by asterisk). HPGDS-ir cells in the stroma most likely represent tissue macrophages (arrowhead in **E**). Again, double-immunofluorescence analysis of ChAT (goat-anti-ChAT antiserum) with HPGDS revealed co-expression in these cells (**D**a–c, **E**a–c, and **F**a–c). FLAP-ir is present in solitary cells in the human small intestine mucosal epithelium (**G**, marked with asterisks), and in pancreatic ducts (**I**, duct marked by asterisks). FLAP-ir is not present in the peribiliary gland epithelium (**H**). Here, presumably epithelium-associated macrophages stain positive for FLAP (arrowhead in **H**). Double-immunofluorescence analysis of villin with FLAP revealed co-expression in small intestine (**G**a–c), and in pancreatic ducts (**I**a–c), but not in peribiliary glands (**H**a–c). The single antibody labeling (a,b) is shown in greyscale, the composite (**c**) in green (ChAT, Villin) and in red (COX1, HPGDS, FLAP) color coding, respectively. The bar in **A** equals 20 µm and applies to all bright-field images. The bar in **Aa** equals 10 µm and applies to all images from immunofluorescence analysis.

Tuft cells in the mouse intestinal epithelium express the key enzymes needed for the production of prostaglandin D2 (PGD2), namely COX-1 and HPGDS^5,14,21^. In our study, COX1-ir was also present in solitary slim cells in our samples from the human small intestine (Fig. 4A), as well as in solitary cells in the epithelia of human peribiliary glands (Fig. 4B) and pancreatic ducts (Fig. 4C). All these cells co-labeled for ChAT-ir (Fig. 4A–Ca–c). Immunohistochemistry for HPGDS resulted in an equivalent staining pattern in small intestine (Fig. 4D), peribiliary glands (Fig. 4E), and pancreatic ducts (Fig. 4F). Again, HPGDS-positive cells were co-ir for ChAT (Fig. 4D–Fa–c). Lipocalin-type PGDS-ir was not detectable in our human samples (data not shown). Only recently, mouse airway and intestinal brush cells were identified as a source of leukotriene production^5,46^. Using antibodies against the 5-lipoxygenase-activating protein, FLAP, we detected solitary cells with a tuft cell morphology in the mucosal linings of the human intestine (Fig. 4G), and in pancreatic ducts (Fig. 4I). Within the peribiliary gland epithelium FLAP-ir cells did not localize to the epithelium, but were flat-associated to the basal membrane (Fig. 4H). Co-staining of FLAP with villin revealed that FLAP-ir cells represent tuft cells in the small intestine (Fig. 4Ga–c), and in pancreatic ducts (Fig. 4Ia–c), but staining was mutually exclusive in peribiliary glands (Fig. 4Ha–c).

### Cholinergic tuft cells are separate from the enteroendocrine cell population

**Figure 5:**
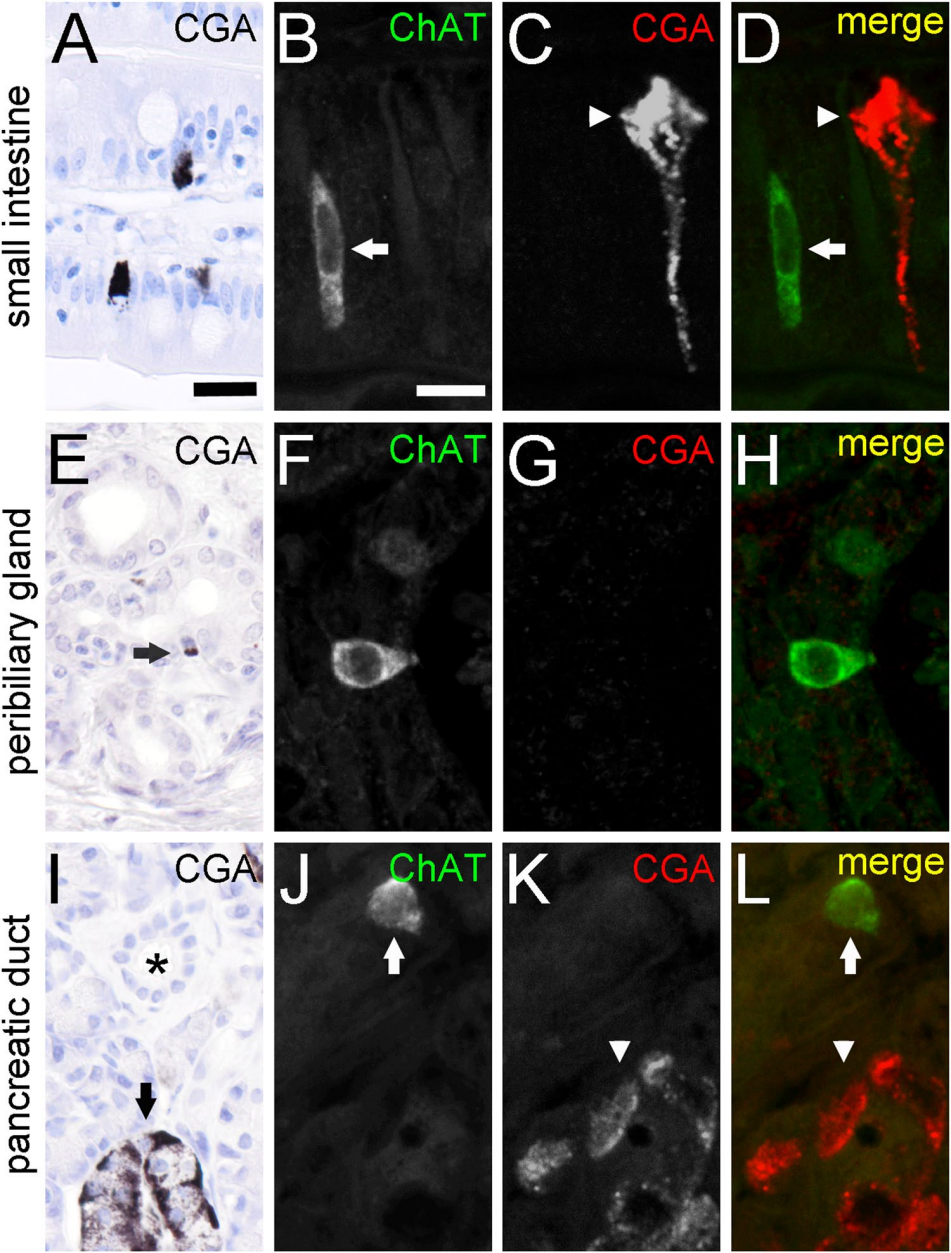
ChAT-immunoreactive tuft cells are separate from CGA-immunoreactive enteroendocrine cells. CGA-ir is present in enteroendocrine cells of the small intestine (**A**). Cell staining is most prominent at the basal cell pole. Double-immunofluorescence analysis of ChAT (goat-anti-ChAT antiserum, arrow) with CGA (arrowhead) revealed non-co-existence (**B–D**). CGA-ir is mostly absent from extrahepatic peribiliary gland epithelium (**E**). Only rarely, single cells show a basally located weak staining (arrow in **E**). Again, co-immunofluorescence analysis revealed non-co-existence of ChAT with CGA (**F–H**). In the pancreas, CGA-ir is present in cells of the islands of Langerhans (**I**, marked by arrow), but is absent from intra- and interlobular ducts (asterisk in **I**). Hence, ChAT (arrow) and CGA (arrowhead) immunoreactions do not co-localize in double-immunofluorescent analysis (**J–L**). The single antibody labeling is shown in greyscale, the composite in green (ChAT) and red (CGA) color coding. The bar in **A** equals 20 µm and applies to all bright-field images. The bar in **B** equals 10 µm and applies to all images from immunofluorescence analysis.

Finally, we asked if also enteroendocrine cells are a source of ChAT expression, as previously suggested^14^. However, we found that CGA, a marker protein of the gut enteroendocrine cell lineage^47^, was absent from ChAT-ir cells in the intestinal (Fig. 5A–D), accessory bile duct (Fig. 5E–H), and pancreatic duct (Fig. 5I–L) epithelia.

## Discussion

The present study provides the first characterization of the cholinergic tuft cell system in the human gastro-entero-pancreatic and extrahepatic biliary tract. Immunohistochemistry for the cholinergic marker enzyme, ChAT, revealed that ChAT not only serves as a reliable tuft cell marker throughout the human entero-pancreatic and extrahepatic biliary systems, but also highlights the central role ACh may play in the diverse functions that recently have been assigned to tuft cells. Co-expression of ChAT with additional tuft cell biomolecule marker (COX1, HPGDS, FLAP) provides a basis for the presumed immune-regulatory role these cells play in health and disease.

The distribution patterns of ChAT-positive tuft cells in the human small and large intestines as demonstrated here essentially correspond to that reported for rodent intestines^21^. Hence, solitary ChAT-positive tuft cells reside to both, crypts and villi of the small and large intestine. A restriction of cholinergic tuft cells to the lower villus and crypt compartments in human ileum and colon is suggestive of a faster turnover of these cells in these regions as compared to duodenum and jejunum. ChAT-positive tuft cells were not present in our samples from human stomach. This is in contrast to the reported abundance of ChAT-positive tuft cells in the columnar epithelium at the squamo-columnar junction, and in other parts of the gastric mucosa of rodents^21,48,49^. The absence of a limiting ridge region in the human stomach may explain the lack of a bona fide cholinergic tuft cell population in the human gastric mucosa, albeit human gastric epithelial cells exhibiting immunohistochemical staining for DCLK1 or COX2 in the context of inflammation, hyperplasia, and cancerogenesis have been reported^31,48,49^. Thus, the existence of a human gastric tuft cell subpopulation that lacks a cholinergic phenotype has to be considered. Also, our observations of a presence (small and large intestine), or absence (stomach) of cholinergic tuft cells may not account for the entire organ, and sub-regional variations may exist.

A cholinergic tuft cell phenotype has not been reported yet for the human biliary tract, in spite of ultrastructural evidence that tuft cells are present^39^. We found ChAT immunoreactive tuft cells scattered throughout extra-hepatic peribiliary glands, while they were absent from the gall bladder and common bile duct epithelium. Such a rather limited distribution pattern is in contrast to rodents, where numerous cholinergic tuft cells are present throughout the gall bladder, especially in its neck region, and in the main epithelium of the extra-hepatic bile duct system^21^. Hence, the non-existence of cholinergic tuft cells in our samples from human gall bladder and common bile duct does not exclude a focal presence in distinct microcompartments of these organs. To determine the specific roles of cholinergic tuft cell signaling in health and disease at the particular sites of the human extra-hepatic biliary tree presented in our report warrant further functional and clinical investigations, especially in the context of a potential impact of pancreatic adenocarcinoma on the local tuft cell occurrence (see below).

In the human pancreas, we frequently observed solitary cholinergic tuft cells residing in intra- and interlobular ducts, which extends observations by others^40^. Cholinergic tuft cells were absent from acini, and from the main pancreatic duct. Again, such a distribution pattern seems to be a specialty for humans, since in rats a vast number of tuft cells is present in the main pancreatic duct^11^, while this cell type is completely absent from the normal mouse pancreas^40^. Recently, presence of DCLK1 in the main pancreatic duct of the mouse has been reported^50^, and increased expression of DCLK1 was a feature of human pancreatic adenocarcinoma^51,52^. In addition to being a tuft cell marker in rodents^7^, DCLK1 marks certain tumor-initiating cancer cells^44,53^. DCLK1-positive epithelial cells in normal and diseased human pancreatic tissue^54^ have not been analyzed with regards to a cholinergic co-phenotype, and their origin is under debate. One explanation is that these cells are not bona fide tuft cells, but arise by a process called transdifferentiation from pancreatic acinar cells, and phenocopy tuft cells from the pancreaticobiliary duct^40^. Our detection of cholinergic tuft cells in the normal human pancreatic duct system, together with their biomolecule co-phenotypes (see below), is suggestive of a role in immunological defense rather than cancer.

In our study we identified advillin as a structural tuft cell marker that is superior to the established markers CK18 and villin. The complete and selective co-localization of advillin with ChAT, and the complete segregation of ChAT staining from CGA-positive enteroendocrine cells rules out the possibility that either advillin or ChAT are expressed by enteroendocrine cells, as suggested in other reports^35^. In addition, given the high sequence homology of human villin and advillin it is conceivable that villin antibodies cross-reacted with advillin in tuft cells, rather than monitoring true presence of villin. Nevertheless, since advillin is also a tuft cell-specific marker in the mouse intestinal mucosal epithelium^5^, experimental targeting of tuft cells with the aid of the advillin promotor will be a useful addendum to the existing genetic tools^16^.

We found that intestinal, biliary, and pancreatic cholinergic tuft cells constitutively express two enzymes necessary for prostaglandin D2 production, namely COX1 and HPGDS. This agrees with observations in mice, where a minimal tuft cell core gene signature was defined that included these markers^14,16^, and with an observation in human intestine, where HPGDS immunoreactive tuft cells were identified in adenoma and in adenocarcinoma^14^. Furthermore, using anti-FLAP antibodies in our studies, we found that intestinal and pancreatic cholinergic tuft cells are endowed with leukotriene biosynthesis capacity, while biliary tract tuft cells are not. Alox5 had already been shown previously to be enriched in mouse intestinal tuft cells^5^, and FLAP was detected specifically in mouse airway brush cells^46^. Thus, some tuft cells from the human alimentary tract may secrete a ‘soup’ of bioactive molecules, including ACh, prostaglandins and leukotrienes into their local microenvironment. However, specific functions for these effector molecules derived from tuft cells have not yet been proposed, and the target cells expressing the cognate receptors not been identified.

Numerous experimental studies in rodents have delineated the emerging concept that cholinergic signaling may be an integral part of the functioning of tuft cells in the context of chemosensation along epithelial barriers^23,24,26,48^. Chemosensation by tuft cells requires presence of G-protein-coupled transmembrane receptors at the luminal surface of tuft cells. Binding of proper ligands, e.g. quorum sensing molecules^55^, is assumed to activate the canonical taste transduction cascade, which includes α-gustducin, PLCβ2, and TRPM5. Ultimately, this intracellular signaling cascade may result in a release of ACh at the abluminal, and perhaps lateral surfaces, into the nearby complex microenvironment, which consists of a heterogeneous population of epithelial cells, lamina propria immune and stromal cells, as well as subepithelial nerve endings of sensory afferent and autonomic efferent fibers. If human intestinal, biliary, and pancreatic cholinergic tuft cells express components of the taste transduction cascade, as determined already for human vallate taste buds^56^, requires further investigation. Nevertheless, the presence of markers for immune-regulatory biomolecule production in human alimentary tract tuft cells (see above) suggests that these cells are involved in chemosensation and regulation of a local immune circuitry. Given the demonstrated absence of VAChT from tuft cells in the intestinal, extrahepatic biliary, and pancreatic tracts, the exact mechanism of the presumed release of ACh from tuft cells cannot be by regulated vesicular exocytosis and, therefore, remains enigmatic. Choline transporter-like proteins are alternative candidates, since they have been shown to be involved in non-neuronal ACh metabolism in humans^57^.

The physiological and pathophysiological roles of tuft cell-derived cholinergic signaling in the human entero-pancreatic and biliary systems is a matter of speculation. In any case, ACh liberated from tuft cells upon sensing luminal chemosignals is likely to have actions on the various nearby cellular and neural targets bearing diverse nicotinic and/or muscarinic receptors. In this context, it has to be considered that ACh released from tuft cells would compete with ACh released by vesicular exocytosis from cholinergic nerve endings. The submucous plexus neurons and subepithelial endings have been shown to contain VAChT and to target the epithelial and lamina propria tuft cell microenvironment (Fig. 1)^38^. Another possible source of ACh are subpopulations of T cells that have been shown to code for ChAT^58^. ACh released from tuft cells, alone or together with ACh from local nerve endings and local T cells, can be expected to influence in a paracrine manner diverse epithelial cells, i.e. enterocytes, goblet cells, Paneth cells, enteroendocrine cells, intestinal stem cells, and transit-amplifying progenitor cells. In addition, a wide variety of cells in the lamina propria, i.e. mesenchymal cells, macrophages, innate lymphoid cells type 2 (ILC2s), mast cells, dendritic cells, T cells, B cells, Tregs, mast cells, basophils and eosinophils, all shown to be endowed with nicotinic and/or muscarinic cholinergic receptors^59^, are possible targets of tuft cell derived ACh. Thus, ACh from tuft cells is likely to contribute to cholinergic modulation of diverse modalities such as barrier function, type 2 innate and adaptive immune responses against e.g. helminths, allergic effector cells, and cancerogenesis^44,60^. Whether cholinergic human tuft cells, like cholinergic tuft cells in mice, represent the host reservoir for chronic infection by norovirus^61^ is not yet known. In any case, the precise outcome of a tuft cell-derived cholinergic signaling will definitely depend on the cellular composition of the local microenvironment, and on the disease modality.

## Methods

### Collection and preparation of human tissue samples

Samples from normal human stomach (n = 6, a mean of 25.2 mm^2^ epithelium investigated per section), duodenum (n = 6, 24 mm^2^/section), ileum (n = 6, 34.6 mm^2^/section), colon (n = 6, 12.1 mm^2^/section), and pancreas (n = 5, 67 mm^2^ total organ area/section), were collected by the authors between 1994 and 2000 at Inselspital Hospital (Bern, Switzerland) during multiple organ dissection from 22 (10 male and 12 female) brain-dead organ donors with an average age of 35.5 years (range: 7–74). Samples from jejunum (n = 6, 28.2 mm^2^ epithelium/section), gall bladder (n = 5, 12,75 mm^2^ epithelium/section), and bile ducts (n = 5, 24.4 mm^2^ total organ area/section) were collected between 2005 and 2014 at the University Hospital (Heidelberg, Germany) during pancreaticoduodenectomies performed for pancreatic adenocarcinoma (n = 11) or chronic pancreatitis (n = 2) in 13 (7 male and 6 female) patients with an average age of 65.0 years (range: 49–76).

The procurement of human material during surgery was done in accordance with local regulations, and approved by the Ethics Committees of the Medical Faculties of the University of Bern, Switzerland or Heidelberg, Germany. Informed consent was obtained from all participants and/or their legal guardian/s.

For subsequent immunohistochemical analyses, all human tissues were removed rapidly during surgery and fixed overnight in buffered 4% paraformaldehyde solution and embedded into paraffin after dehydration. Paraffin-embedded specimens were cut in 4 µm sections using a microtome and mounted on silanized glass slides.

### Immunohistochemistry

Single brightfield immunohistochemistry was performed as described previously^21^. In brief, tissue sections were deparaffinized in xylene and rehydrated through a graded series of isopropanol, including 30 min incubation in methanol/0.3% H_2_O_2_ to block endogenous peroxidase activity. Subsequently, antigen retrieval was achieved by incubation in 10 mM sodium citrate buffer (pH 6.0) at 92–95 °C for 15 min. Non-specific binding sites were blocked with 5% bovine serum albumin (BSA) in 50 mM phosphate buffered saline (PBS, pH 7.45) for 30 min, followed by an avidin–biotin blocking step (Avidin–Biotin Blocking Kit, Boehringer, Ingelheim, Germany) for 20 min each. Primary antibodies (see Table 1 for details) were applied in PBS/1% BSA over night at 16 °C followed by 2 h at 37 °C. After several washes in PBS, the sections were incubated for 45 min at 37 °C with species-specific biotinylated secondary antibodies (see Table 1 for details), diluted 1:200 in PBS/1% BSA, washed, and incubated for 30 min with avidin–biotin–peroxidase complex (Vectastain Elite ABC kit; Vector Laboratories, Burlingame, CA). Immunoreactions were then visualized by 8 min incubation in 3,3-diaminobenzidine (DAB, Sigma Aldrich, Deisenhofen, Germany), enhanced by the addition of 0.08% ammonium nickel sulfate (Fluka, Bucks, Switzerland). After three 5 min washes in distilled water, some sections were counterstained with hemalaun solution, dehydrated through a graded series of isopropanol, cleared in xylene and finally mounted under coverslips. Digital bright-field pictures were taken with an Olympus AX70 microscope, equipped with an Olympus UC90 camera and Olympus cellSens analyses software.

**Table-1:** Characteristics of primary and secondary antibodies used for immunohistochemistry.

Primary antibodies						
Antigen	Abbr.	Epitope	Host species	Dilution (BF/IF)	Source	Catalog no./(internal code)
Advillin	AVIL	Recombinant peptide corresp. to amino acids 447 to 532 of human advillin	rabbit	500/50	antibodies-online.com	ABIN4278537
Choline acetyltransferase	ChAT	Human ChAT	goat	100/50	Chemicon	AB144P
Choline acetyltransferase	ChAT	Synthetic peptide (peptide 3), corresponding to aa 168–189 of porcine brain ChAT^62^	rabbit	n.a./100	M. Schemann, Munich, Germany	PO3; Yeboah
Chromogranin A	CGA	Bovine CGA (aa 316–329, WE-14 epitope)^63^	rabbit	30,000/ 3,000	L. Eiden, Bethesda, USA	(Lenny/09)
Cyclooxygenase 1	COX1	Peptide from carboxy terminus of human COX1 (C-20)	goat	2,000/500	Santa Cruz Biotech.	sc-1752
Cytokeratin 18	CK18	Peptide corresponding to C-terminus of human cytokeratin 18.	rabbit	2,000/200	Sigma Aldrich	SAB4501665
Doublecortin-like kinase 1	DCLK1	Peptide corresponding to C-terminus of human DCLK1 (aa 690–720)	rabbit	500/50	Abgent	AP7219b
Arachidonate 5-lipogygenase-activating protein	FLAP	Peptide with sequence C-KTISTTISPLLLIP corresponding to C-terminus of human FLAP	goat	1,500/150	Novus Biol.	NB300–891
Prostaglandin-D-synthase, hematopoietic	HPGDS	Recombinant human HPGDS^64^	mouse	2,500/250	M. Lazarus and Y. Urade, Osaka, Japan	n.a.
Vesicular acetylcholine transporter	VAChT	Human VAChT (12 aa from C-terminus)^38,65,66^	rabbit	5,000/ 500	L. Eiden, NIH, Bethesda, USA	(80153)
Villin	VIL	Peptide corresponding to C-terminus of human villin (aa721–780)	mouse	200/50	Santa Cruz Biotech.	sc-365310
**Secondary antibodies**						
**Antigen**	**Ig size**	**Host species**	**Dilution**	**Conjugate**	**Source**	**Catalog no**.
Rabbit IgG	Whole molecule	Donkey	1:200	Biotin	Dianova	711–065–152
Mouse IgG	Whole molecule	Donkey	1:200	Biotin	Dianova	715–065–151
Goat IgG	Whole molecule	Donkey	1:200	Biotin	Dianova	705–065–147
n.a.	n.a.	n.a.	1:200	Streptavidin-Alexa488	Life Technologies (Molecular Probes)	S11223
Rabbit IgG	Whole molecule	Chick	1:200	Alexa647	Life Technologies (Molecular Probes)	A21443
Mouse IgG	Whole molecule	Chick	1:200	Alexa647	Life Technologies (Molecular Probes)	A21463
Goat IgG	Whole molecule	Donkey	1:200	Alexa647	Life Technologies (Molecular Probes)	A21447

Abbr.: BF = brightfield, IF = immunofluorescence, n.a. = not applicable. Companies: Abgent, San Diego, CA, USA; Dianova, Hamburg, Germany; Life Technologies, Carlsbad, USA; Novus Biologicals, Centennial, CO, USA; Santa Cruz Biotechnologies Inc., Heidelberg, Germany; Sigma-Aldrich, St. Louis, MO, USA. Select references link to studies where the antibody suitability and specificity were proven.

Double immunofluorescence analysis was performed on paraffin sections as follows: After deparaffinization and blocking procedures (see above), two primary antibodies from different donor species (see Table 1 for details) were co-applied in PBS/1% BSA and incubated over night at 16 °C, followed by 2 h at 37 °C. After extensive washing in distilled water followed by PBS, immunoreactions for one antibody were visualized with a secondary antibody labeled with Alexa Fluor 647. The other antibody was visualized by a two-step procedure using a species-specific biotinylated secondary antibody, followed by streptavidin conjugated with Alexa Fluor 488 (see Table for details). Incubation times were 45 min with the biotinylated secondary antibody only, followed by 2 h incubation with a mixture of fluorochrome-conjugated secondary antibody and streptavidin.

Immunofluorescence signals were documented in a surface scan using a BX50WI confocal laser scanning microscope (Olympus Optical, Hamburg, Germany) and Olympus Fluoview 2.1 software, and stored as false color images (8-bit tiff format) without modifications in brightness and contrast.
